# Dissecting the fate of *Foxl2*-expressing cells in fetal ovary using lineage tracing and single-cell transcriptomics

**DOI:** 10.1038/s41421-022-00492-1

**Published:** 2022-12-27

**Authors:** Jingjing Zhou, Xiangxiang Jiang, Haowei Wu, Lianjun Zhang, Min Chen, Min Chen, Zhiming Shen, Xudong Guo, Hongmei Wang, Fei Gao

**Affiliations:** 1 State Key Laboratory of Stem cell and Reproductive Biology, Institute of Zoology, Chinese Academy of Sciences, Beijing, China; 2 Institute for Stem Cell and Regeneration, Chinese Academy of Sciences, Beijing, China; 3 University of Chinese Academy of Sciences, Beijing, China; 4 The Collaborative Innovation Center, Jining Medical University, Jining, Shandong China; 5 NHC Key Laboratory of Study on Abnormal Gametes and Reproductive Tract, Anhui Medical University, Hefei, China; 6 Department of Hematological Malignancies Translational Science, Hematologic Malignancies and Stem Cell Transplantation Institute, Beckman Research Institute, City of Hope National Medical Center, Duarte, CA USA; 7 Guangdong and Shenzhen Key Laboratory of Male Reproductive Medicine and Genetics, Institute of Urology, Peking University Shenzhen Hospital, Shenzhen Peking University-The Hong Kong University of Science and Technology Medical Center, Shenzhen, China; 8 State Key Laboratory of Reproductive Regulation & Breeding of Grassland Livestock, Inner Mongolia University, Hohhot, China

**Keywords:** Developmental biology, Cell biology

## Abstract

Gonad somatic cells acquire sex-specific fates during sex determination. In XX gonad, a subset of somatic cells expresses *Foxl2* after sex determination which is considered the progenitor of granulosa cells. However, whether these cells also contribute to other cell types at later developmental stages is unknown. In the present study, the cell fate of *Foxl2*-expressing cells in fetal ovaries was analyzed by lineage tracing and single-cell transcriptomics. We found that *Foxl2*-expressing cells gave rise to three cell types at later developmental stages, including granulosa cells, theca-interstitial cells, and stromal cells. Series single-cell RNA sequencing revealed FOXL2-positive cells were divided into two clusters at P0. One group further differentiated into granulosa cells and Theca-G (Theca-interstitial cells derived from granulosa) at P14. Another group was classified as stromal cell lineage, then a small portion of them further differentiated into *3β-HSD*-positive Theca-S (Theca-interstitial cells derived from stroma). *Cyp17a1* was expressed in Theca-S, but not in Theca-G. This study demonstrated that *Folx2*-expressing cells in XX gonad after sex determination are multipotent and theca-interstitial cells are derived from different progenitors. Our data provided an important resource, at single-cell resolution, for a better understanding of somatic cell differentiation in ovary development.

## Introduction

In mammals, both testes and ovaries are derived from the genital ridge, which forms as a thickening of the epithelial layer on the ventromedial surface of the mesonephros^1,2^. Mammalian sex is determined in the fetal gonad by the presence or absence of the Y-linked high mobility group (HMG) domain transcription factor *Sry* gene^3^. *Sry* directs the Sertoli cell differentiation in XY gonads by inducing *Sox9* expression^3–5^. In XX gonads, which lack *Sry* expression, the somatic cells express FOXL2 under the regulation of the RSPO1/WNT4-β-catenin (CTNNB1) signaling pathway. These cells are considered the progenitors of granulosa cells and differentiate into granulosa cells in developing ovarian follicles at later developmental stage^6–8^. Forkhead box L2 (*FoxL2*) encodes the forkhead transcription factor 2, which is a member of the winged helix/forkhead transcription factors^9^ and plays an essential role in female reproduction. Mutations of the *Foxl2* gene in humans are associated with Blepharophimosis Ptosis Epicanthus Inversus Syndrome (BPES). One of the phenotypes associated with type 1 BPES is a premature ovarian failure in females^10^. Deletion of *Foxl2* leads to ovary dysgenesis and infertility in female mice. *Foxl2* mutant granulosa cells are unable to undergo the squamous-to-cuboidal transition, which in turn causes progressive follicular depletion and infertility^11,12^.

Leydig cells and theca-interstitial cells are steroidogenic cells in male and female gonads, respectively. The steroid hormones produced by steroidogenic cells play essential roles in the development of germ cells and secondary sexual characteristics. Leydig cells first appear in testes at E12.5 (12.5 days post coitus), whereas theca-interstitial cells are observed postnatally in the ovaries along with the formation of primary follicles. The origins of Leydig cells^13,14^ and theca-interstitial cells^15^ have been investigated previously. It has been reported that theca-interstitial cells in the ovary are derived from two sources: WT1-positive cells indigenous to the ovary and GLI1-positive mesenchymal cells that migrate from the mesonephros^15^. Our previous studies also demonstrate that steroidogenic cells in female gonads are derived from WT1-positive cells^16^. Our recent study finds that the inactivation of WT1 in FOXL2-positive cells also causes these cells to transform into 3β-HSD-positive steroidogenic cells^17^. These results suggest that FOXL2-positive cells in fetal gonads have the potential to develop into steroidogenic cells.

In the present study, the cell fate of FOXL2-positive cells in the fetal ovary was examined by lineage tracing and single-cell transcriptomics. We found that other than granulosa cells, these cells also gave rise to theca-interstitial cells and ovarian stromal cells. Series single-cell RNA sequencing revealed that a subset of theca-interstitial cells (Theca-G) were directly differentiated from granulosa cell lineage, whereas other 3β-HSD-positive theca-interstitial cells (Theca-S) were derived from ovarian stromal cells which were separated from granulosa lineage at P0(postnatal day 0). We also found that Theca-S were CYP17A1-positive, whereas Theca-G did not express CYP17A1.

## Results

### GFP signal was detected in both granulosa cells and theca-interstitial cells of mT/mG; Foxl2-Cre mice

**Fig. 1:**
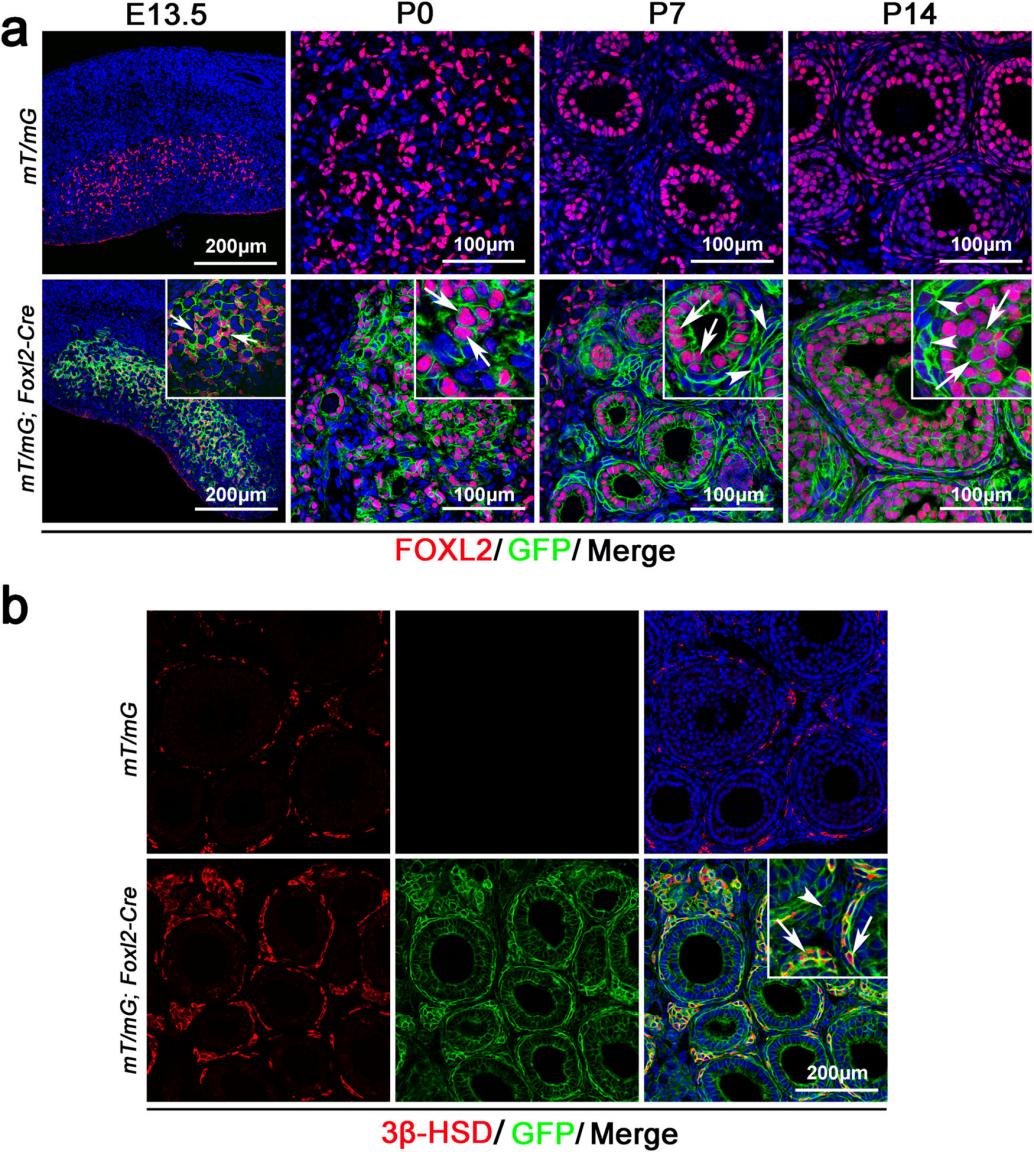
Lineage tracing of *Foxl2*-expressing cells in the fetal ovary with *Foxl2*-*Cre* mice. **a** *Foxl2*-*Cre* mice were crossed with *mT/mG* reporting mice and GFP signal was examined by immunostaining. GFP signal was not detected in the ovaries of *mT/mG* mice at E13.5, P0, P7, and P14 (top panel). GFP signal was detected in the ovaries of *mT/mG; Foxl2-Cre* mice at E13.5 (white arrows) and P0 (white arrows). GFP signal was not only detected in FOXL2-positive granulosa cells at P7 (white arrows) and P14 (white arrows) in *mT/mG; Foxl2-Cre* mice, but also in FOXL2-negative theca-interstitial cells (white arrowheads). **b** No GFP signal was detected in the ovaries of *mT/mG* mice at P14 (top panel). 3β-HSD (red) and GFP double positive cells were observed in the ovaries of *mT/mG; Foxl2-Cre* mice at P14 (inset, white arrows). GFP-positive and 3β-HSD-negative (inset, white arrowheads) stromal cells were also observed in *mT/mG; Foxl2-Cre* mice.

To trace the fate of *Foxl2*-expressing cells in fetal ovaries, *mT/mG* reporting mice were crossed with *Foxl2*-Cre mice and the expression of GFP was examined at different developmental stages. *mT/mG* reporting mouse is a double-fluorescent Cre reporter mouse that expresses membrane-targeted tandem dimer Tomato (mT) prior to Cre-mediated excision and membrane-targeted green fluorescent protein (mG) after activation of cyclization recombination enzyme (CRE).^18^ To test *mT/mG; Foxl2*-Cre mouse model, an immunofluorescence experiment was performed in the ovaries of *mT/mG; Foxl2-Cre* mice at different developmental stages. In *mT/mG* ovaries, all the cells were Tomato positive (red), including FOXL2-positive (green) granulosa cells (Supplementary Fig. S1a, top panel). In *mT/mG; Foxl2-Cre* ovaries, no Tomato signal (red) was detected in FOXL2-positive (green) granulosa cells (Supplementary Fig. S1a, bottom panel), indicating that Tomato signal was turned off in Cre-expressing cells. As shown in Supplementary Fig. S1b, no GFP signal was detected in the ovaries of *mT/mG; Foxl2-Cre* mice at E11.5, indicating that *Foxl2-Cre* was not activated at this stage. FOXL2 signal (red) was detected in *mT/mG; Foxl2-Cre* mice at E12.5, while no GFP signal was detected, indicating that *Foxl2-Cre* was not activated at E12.5. A large number of FOXL2 and GFP double positive cells were detected in *mT/mG; Foxl2-Cre* mice at E13.5 (Supplementary Fig. S1b, inset, white arrows), indicating that *Foxl2-Cre* was activated at this stage. We also found some cells surrounded by the GFP signal were negative for the FOXL2 signal (Supplementary Fig. S1b, inset, white arrowheads). Based on the nuclear morphology, these cells are most likely germ cells, not somatic cells. Since the germ cells and somatic cells were mixed together at this stage, the membrane-targeted GFP signal was most likely from adjacent somatic cells. To further confirm this result, MVH and GFP co-immunostaining was performed at E13.5 (Supplementary Fig. S1c). The germ cells were labeled with MVH (red), and no GFP signal was detected in control ovaries (Supplementary Fig. S1c, top panel). In *mT/mG; Foxl2-Cre* mice, some MVH-positive germ cells surrounded by GFP signal were observed (Supplementary Fig. S1c, inset, white arrows). Given the fact that *Foxl2-Cre* is only activated in somatic cells, we speculated that these GFP signal was located at the membrane of adjacent somatic cells. In the ovaries of control *mT/mG* mice from E13.5 to P14(postnatal 14 days), no GFP signal was detected (Fig. 1a). In *mT/mG; Foxl2-Cre* mice, a GFP signal was detected in the gonad somatic cells at E13.5 and the GFP signal was co-localized with FOXL2 protein (Fig. 1a), indicating that GFP signal was specifically activated in *Foxl2*-expressing cells of fetal gonads. GFP signal was continually expressed in FOXL2-positive cells at P0. Interestingly, not only the FOXL2-positive granulosa cells were GFP-positive at P7 and P14, GFP signal was also observed in many FOXL2-negative cells. To further confirm this result, GFP was co-stained with WT1, which is specifically expressed in granulosa cells. Consistent with FOXL2 staining, a GFP signal was detected in both WT1-positive granulosa cells and WT1-negative somatic cells (Supplementary Fig. S2a). Based on the location and morphology, these WT1-negative cells were most likely theca-interstitial and stromal cells. To verify the cell identity, we stained GFP with steroidogenic enzyme 3β-HSD. As shown in Fig. 1b, GFP and 3β-HSD double positive cells were observed in the ovaries of *mT/mG; Foxl2-Cre* mice at P14, indicating that these cells were theca-interstitial cells which were responsible for steroid hormone production in female gonads. However, a small portion of GFP^+^3β-HSDˉ cells were also observed in the interstitium of *mT/mG; Foxl2*-Cre ovaries at P14. These results indicated that FOXL2-positive cells in fetal ovaries might give rise to WT1-positive granulosa cells, 3β-HSD-positive theca-interstitial cells, and 3β-HSD-negative stromal cells.

**Fig. 2:**
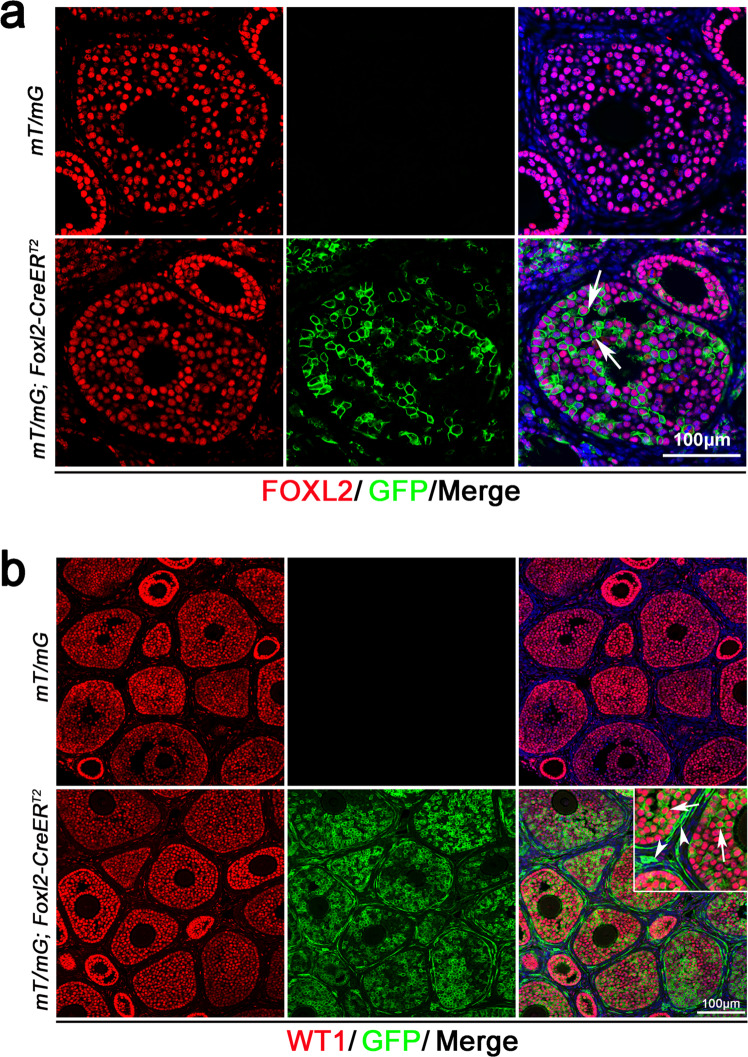
Lineage tracing of *Foxl2*-expressing cells in the fetal ovary with *Foxl2-CreER*^*T2*^ mice. **a** No GFP were observed in theca-interstitial cells when *Foxl2-CreER*^*T2*^ mice was induced with tamoxifen at 3 weeks of age. *mT/mG; Foxl2-CreER*^*T2*^ mice were induced with tamoxifen at 3 weeks of age and the GFP signal was examined by immunostaining. No GFP signal was detected in the ovaries of *mT/mG* mice after tamoxifen induction. GFP signal was only detected in FOXL2-positive granulosa cells (bottom panel, white arrows) of *mT/mG; Foxl2-CreER*^*T2*^ mice and no GFP signal was detected in theca-interstitial cells. **b** GFP-positive cells were observed as both granulosa cells and theca-interstitial cells of *mT/mG; Foxl2-CreER*^*T2*^ mice when Cre was induced with tamoxifen at E13.5. *mT/mG; Foxl2-CreER*^*T2*^ mice were induced with tamoxifen at E13.5 and GFP signal was examined by immunostaining at 3 weeks of age. No GFP signal was detected in the ovaries of *mT/mG* mice after tamoxifen induction (top panel). GFP signal was detected in both WT1-positive granulosa cells (inset, white arrows) and WT1-negative theca-interstitial cells (inset, white arrowheads).

It has been reported that a low level of FOXL2 is also expressed in theca-interstitial cells^11^. To exclude the possibility that the GFP signal detected in theca-interstitial is due to the ectopic *Foxl2* expression, we crossed *mT/mG* mice with *Foxl2-CreER*^*T2*^ mice and Cre activity was induced by Tamoxifen injection at 3 weeks of age. As shown in Fig. 2a, the GFP signal was exclusively detected in FOXL2-positive granulosa cells, not in theca-interstitial and stromal cells. By contrast, when Cre activity was activated at E13.5, a GFP signal was detected in both granulosa cells and theca-interstitial cells at 3 weeks of age (Fig. 2b). Furthermore, FOXL2 and GFP co-immunostaining was performed with P0 ovary of *mT/mG; Foxl2-creER*^*T2*^ mice. As shown in Supplementary Fig. S2b, FOXL2^˗^GFP^+^ (Supplementary Fig. S2b, inset, white arrows) and FOXL2^+^GFP^+^ (Supplementary Fig. S2b, inset, white arrowheads) were detected, indicating that FOXL2-positive cells have been separated into two groups at P0. These results further confirmed that FOXL2-positive cells in fetal gonads not only contributed to granulosa cells, but also other cell populations at later developmental stages.

### The progeny of *Foxl2*-expressing cells was classified into ten transcriptionally distinct cell populations along with ovary development

**Fig. 3:**
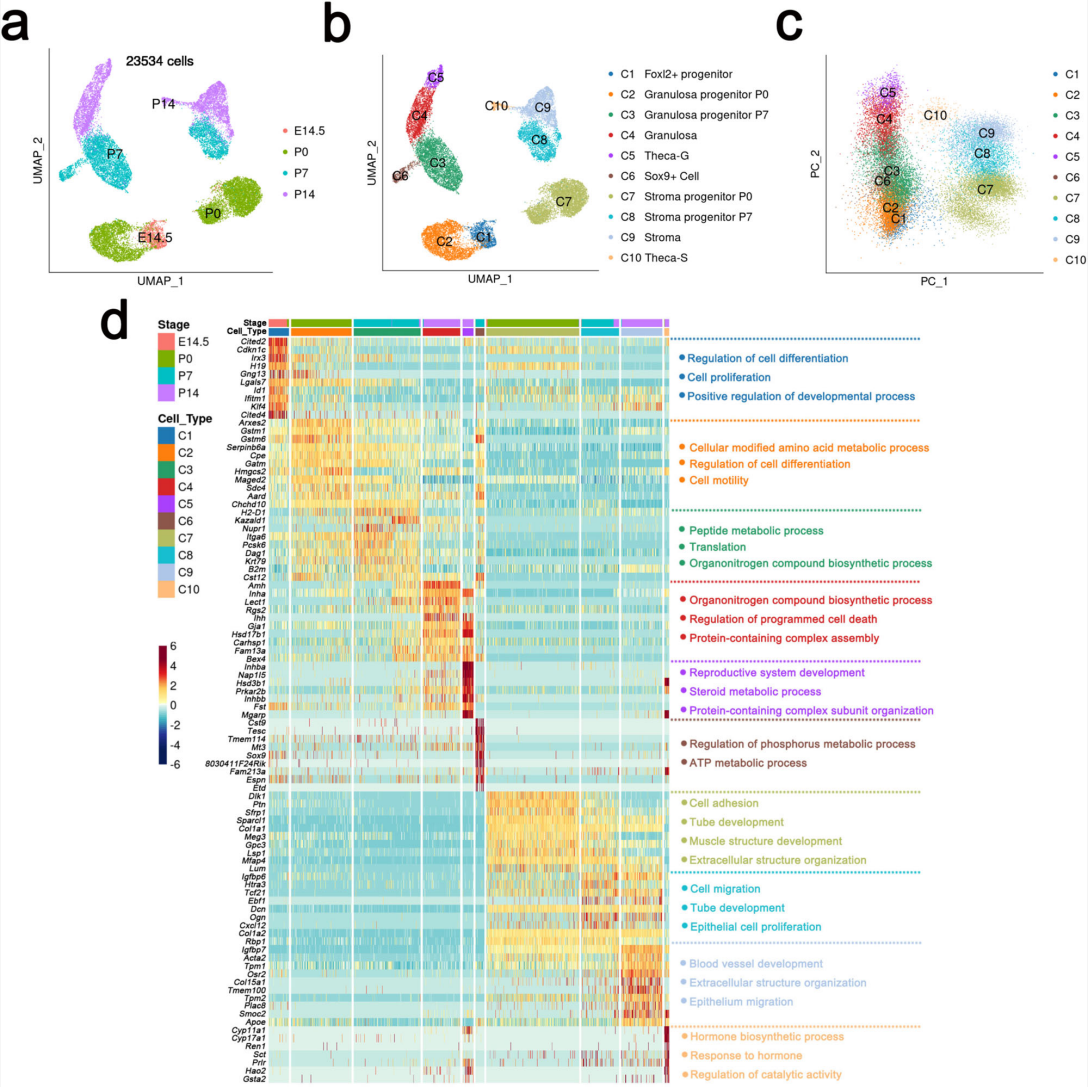
Classification and Identification of progeny of *Foxl2*-expressing cells during ovarian development. **a** Expression matrix-based Uniform manifold approximation and projection (UMAP) plot showed the filtered FOXL2^+^ progenitor-derived cells (23,534 cells). Cells were colored by time points of sample collection. **b** UMAP plot as shown in **a**, with cells colored by cell clusters after unsupervised clustering. **c** Expression matrix-based principle component (PC) plot of cell clusters in **b**. **d** Left, heatmap showing top ten highly expressed genes for each cell cluster shown in **b**, red color corresponded to high expression levels; blue color corresponded to low expression levels. Right, representative enriched items of gene ontology (GO) biological process for the genes highly expressed in corresponding cell clusters.

To characterize and reconstruct the differentiation of *Foxl2*-expressing cells as ovary development proceeds, we crossed *mT/mG* reporting mice with *Foxl2-CreER*^*T2*^ mice and *Foxl2*-expressing cells were labeled by injecting Tamoxifen at E13.5. GFP-positive cells were isolated at E14.5, P0, P7, and P14 by Flow Cytometry. Single-cell RNA-sequencing experiment was performed using the 10× Chromium system. 23,534 GFP-positive cells passed stringent filtering (methods), with a median of ~2300 genes detected per cell (Supplementary Fig. S3). GFP-positive cells present in the developing ovaries were classified based on the highly variable genes, the time points of sample collection and the principle component analysis (Fig. 3a–c). We obtained ten cell clusters (C1 to C10) combining different developmental stages (Fig. 3b, c).

Partition-based graph abstraction and pseudotime reconstruction showed the relationship and the developmental process of these 10 cell clusters (Fig. 3a–c). Expression enrichment of known markers and differentially expressed genes (DEGs) allowed us to assign the identity of the cell clusters and enriched items of gene ontology (GO) biological process for corresponding genes, indicating the biological process of C1 to C10 (Fig. 3d).

### *Foxl2*-expressing progenitor cells were separated into two lineages during ovary development

**Fig. 4:**
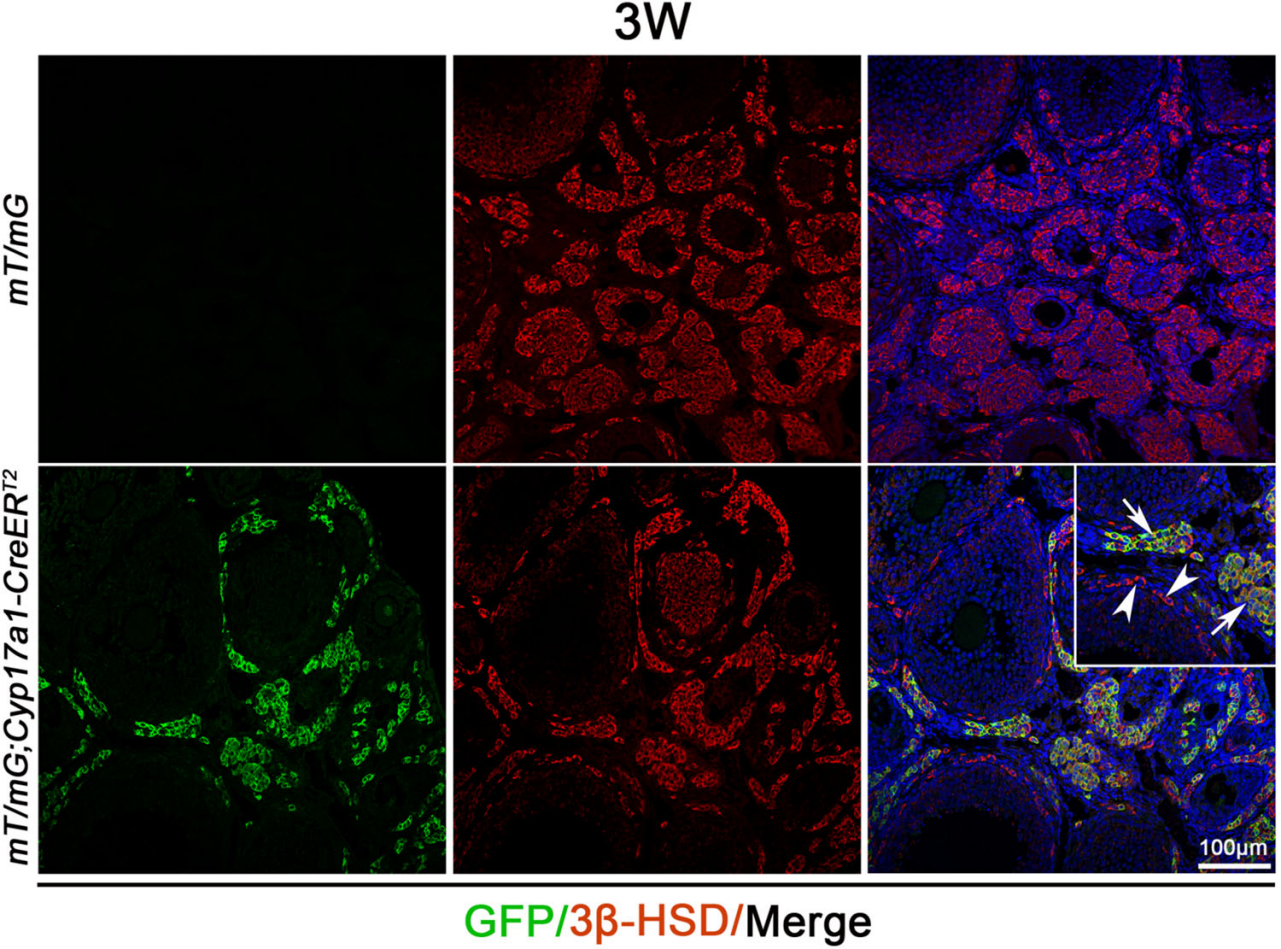
GFP signal was detected in the theca-interstitial cells of *mT/mG; Cyp17a1-CreER*^*T2*^ mice. Cre activity in *mT/mG; Cyp17a1-CreER*^*T2*^ was induced with tamoxifen at 3 weeks of age and GFP signal was examined by immunostaining. 3β-HSD signal was detected in both Theca-G (inset, white arrowheads) and Theca-S (inset, white arrows) and no GFP signal was detected in the ovaries of control mice after tamoxifen induction (top panel).

C1 represented the early progenitor cell population with cells mainly from E14.5 (Fig. 3a, b). The GFP-positive cells from the P0 ovary were classified into two groups, C2 and C7 (Fig. 3a, b). C3, C6, and C8 were from P7 ovaries (Fig. 3a, b). The GFP-positive cells from P14 ovaries were classified into four groups, C4, C5, C9, and C10 (Fig. 3a, b). Principle component analysis (PCA) result indicated that C1–C5 and C7–C10 belonged to two different branches (Fig. 3c). Partition-based graph abstraction (PAGA) plot showed the relationship and trajectories of those 10 cell clusters. C1, C2, C3, C4, and C5 were closely related, whereas C7, C8, C9, and C10 were closely related (Fig. 4a). The pseudotime trajectories reconstructed by monocle3 further indicated the developmental process of cell clusters from C1 to C5 and C7 to C10.

**Fig. 5:**
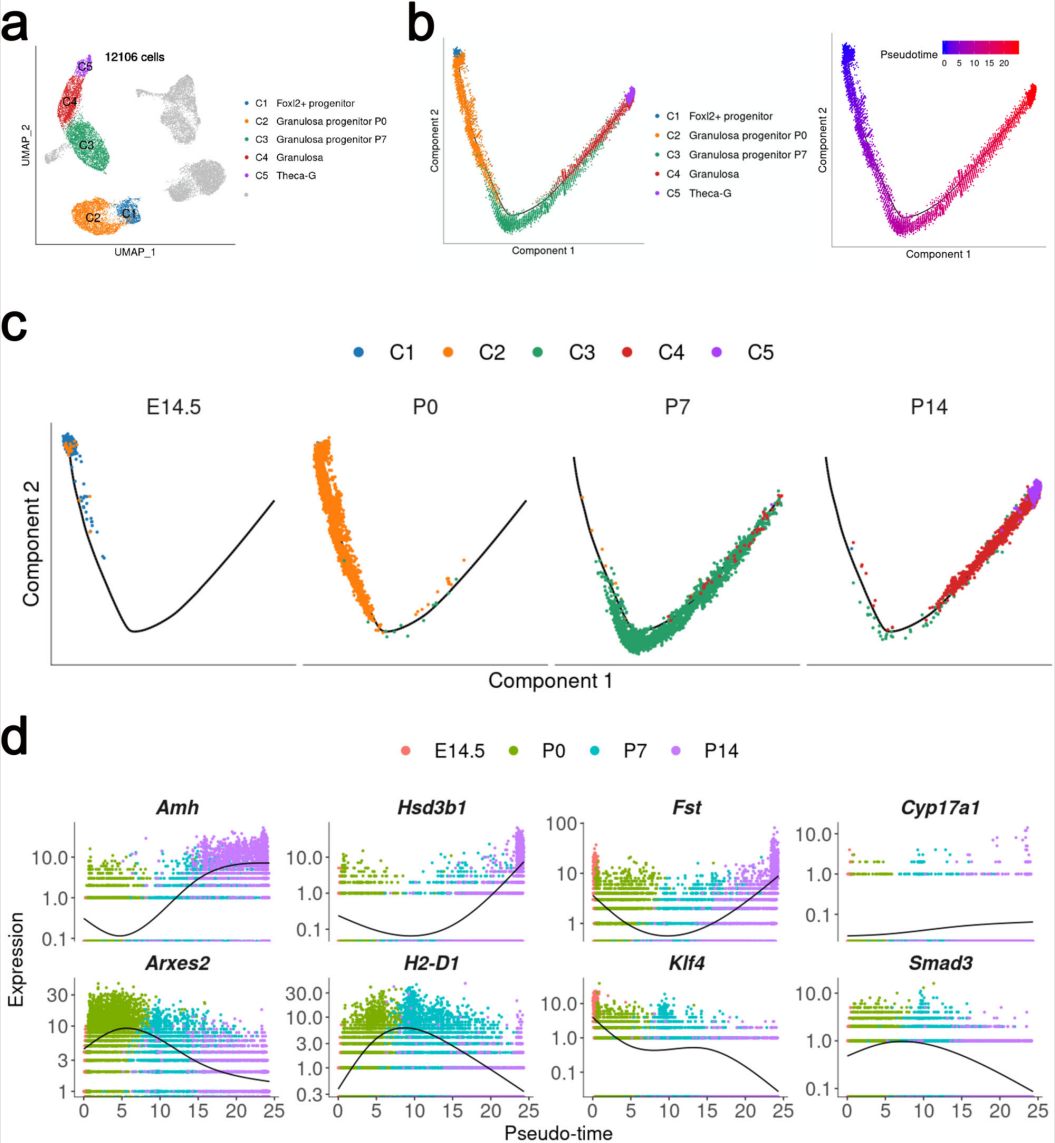
Cell lineage and pseudotime prediction of divergence of granulosa and Theca-G lineages from a common progenitor population. **a** UMAP plot as shown in **3a**, only the granulosa cell lineage (C1–C5, 12,106 cells) was used for the following pseudotime analysis. **b** Pseudotime ordering of cells shown in **a**, with cells colored by cell clusters (left) and pseudotime (right). The color of pseudotime from blue to red, represented the cell states from naïve to mature. **c** Pseudotime trajectory plot from **b** (left) was split by sampling time points, with cells colored by indicated cell clusters. **d** Pseudotemporal kinetics plots showed the highly expressed genes in granulosa and Theca-G cells.

Granulosa cell-specific genes such as *Amh*^19^, *Fst*^20^, *Inha*, and *Ihh*^21^ were expressed in C4 (Figs. 3d, 5d and Supplementary Fig. S6a), indicating this cluster was granulosa cells. *Amh*, *Inha*, and *Fst* were also expressed in C2 and C3, but the expression levels were lower than those in C4 (Fig. 3d and Supplementary Fig. S6a). The results of pseudotime prediction and lineage reconstruction also showed that the cells in C4 were derived from C2 and C3. Based on these results, we speculated that C2, C3, and C4 belonged to the granulosa cell lineage, and C2 and C3 were the intermediate stages of pre-granulosa cells. *Arxes2*, *Gstm1*, *Gstm6, Serpinb6a*, etc, were abundantly expressed in C2 and the top expressed genes in C3 were *H2-D1*, *Chchd10*, *Kazald1*, *Nupr1*, Itga6, etc (Fig. 3d and Supplementary Fig. S6a). However, the functions of these genes in granulosa cell development have not been reported previously.

GO analysis based on highly expressed genes in each cell cluster indicated that the early progenitor cells were mainly proliferating cells (C1, cell proliferation, and regulation of cell differentiation). The biological processes of “organonitrogen compound biosynthetic process”, “peptide metabolic process” and “regulation of cell differentiation” were mostly enriched in pre-granulosa cells (C2 and C3) and granulosa cells (C4) (Fig. 3d). C5 was closely related to C4 and both of them came from P14 ovaries (Fig. 3a–c). High level of steroidogenic enzyme genes, *Hsd3b1* and *Hsd17b1*, were expressed in this cluster (Fig. 3d and Supplementary Fig. S6a), indicating that C5 were theca-interstitial cells which were most likely derived from granulosa cell lineage (Theca-G). C6 was closely related to C3, but the cell number was much less. Surprisingly, Sertoli cell-specific gene *Sox9* was detected in this cluster (Fig. 3d). The results of immunohistochemistry also showed that a small number of cells in ovaries at P7 were Sox9-positive (Supplementary Fig. S4, inset). The exact functions of these cells were unclear and need further investigation.

**Fig. 6:**
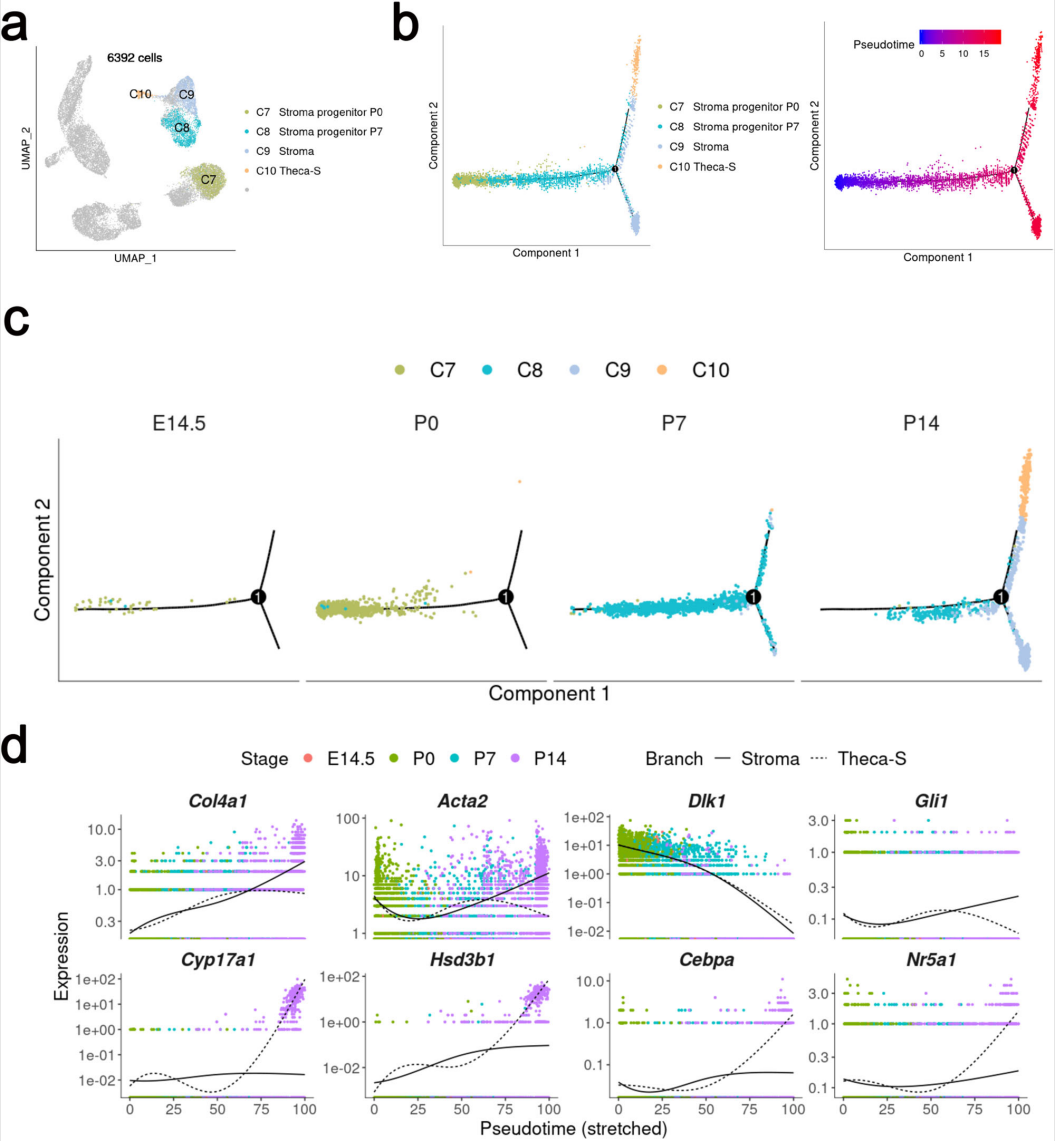
Cell lineage and pseudotime prediction of the divergence of stromal and Theca-S lineages from a common progenitor population. **a** UMAP plot as shown in **3a**, only the non-proliferating cells (C7–C10, 6392 cells) were used for the following pseudotime analysis. **b** Pseudotime ordering of cells shown in **a**, with cells colored by cell clusters (left) and pseudotime (right). Color of pseudotime from blue to red, represented the cell states from naïve to mature. **c** Pseudotime trajectory plot from **b** (left) was split by sampling time points, with cells colored by indicated cell clusters. **d** Pseudotemporal kinetics plots showed the highly expressed genes in stromal and Theca-S cells.

Stromal cell-associated genes *Ptn*^22^, *Sfrp1*^23^, *Col1a1*^24^, and *Mfap4*^25^ were abundantly expressed in C7 (Fig. 6d and Supplementary Fig. S6a). The cells in C8 and C9 also expressed stromal cell-associated genes, such as *Col1a1* and *Mfap4* (Figs. 3d, 6d and Supplementary Fig. S6a). Thus, C7–C9 were annotated as stroma progenitors and stromal cells. The stroma progenitor-expressing genes related to the morphological organization (C7 and C8, “extracellular matrix organization”, “cell migration”, “cell adhesion”, and “tube development”) were enriched in these cell clusters. Cluster 10 was developmentally related to Cluster 9 and also came from P14 ovaries. Interestingly, high levels of steroidogenic enzyme genes, *Hsd3b1* and *Cyp11a1*, were expressed in C10. Based on gene expression, cluster 10 was also annotated as theca-interstitial cells, which were most likely derived from stroma cell lineage (Theca-S).

### Theca-G and Theca-S were derived from different progenitor cells and gene expressing was different

Based on gene expression, both C5 and C10 were theca-interstitial cells. However, C5 was derived from granulosa cells lineage (Theca-G), and still expressed granulosa-specific genes, such as *Fst* and *Ihh* (Supplementary Fig. S5d). C10 was differentiated from stroma cell lineage (Theca-S), and still expressed stromal cell-associated genes, such as *Igfbp7* and *Smoc2* (Supplementary Fig. S5e). Meanwhile, *Cyp17a1* was exclusively expressed in C10, and Inhba was specifically expressed in C5 (Supplementary Fig. S5d, e). To further identify these two groups of steroidogenic cells, *mT/mG* reporting mice were crossed with *Cyp17a1-CreER*^*T2*^ mice and Cre activity was induced by Tamoxifen induction at 3 weeks of age. As shown in Fig. 4, the GFP signal was detected in the 3β-HSD-positive theca-interstitial cells (Fig. 4, inset, white arrows), which were located in the interstitium, but not in the 3β-HSD-positive theca-interstitial cells (Fig. 4, inset, white arrowheads) which surrounded the ovarian follicles. These results suggested that 3β-HSD-positive theca-interstitial cells with different locations in the ovary were derived from different progenitor cells and the gene-expressing profiles were also different.

### The development of granulosa cell and stromal cell lineage

To further confirm our results, pseudotime prediction was conducted. For granulosa lineage, C1–C5 cells were selected to construct pseudotime trajectory (Fig. 5a). Pseudotime trajectories showed the differentiation of granulosa and Theca-G cells (Fig. 5b, c). There was no divergence in the pseudotime trajectories of the granulosa lineage, and C5 (Theca-G) appeared later than C4 (granulosa), indicating that Theca-G was differentiated from granulosa. Pseudotime profiles were scrutinized based on genes are known to be expressed in granulosa (*Amh*, *Fst)* and theca cells (*Hsd3b1*). The gene expression profile showed that *Amh* expression was increased from E14.5 to P14 in the granulosa cell lineage (Fig. 5d). Interestingly, we found that the expression of *Klf4* was significantly decreased from E14.5 to P14. The expression of *Arxes2* and *H2-D1* was increased first, then decreased in this lineage (Fig. 5d), suggesting that these genes are probably also involved in the regulation of granulosa cell differentiation. Expression of genes specific to granulosa (C4) and Theca-G (C5) was skewed preferentially toward the beginning and end of the trajectories (Fig. 5d). In this process, *Smad3*, *Nfe2I1*, *Mef2d*, *Rreb1*, *Foxp4*, et al., were highly activated in C3 at P7, which indicated that these transcription factors could regulate the differentiation of granulosa and Theca-G (Supplementary Figs. S5f–i). *Tcf7* was essential to the cell fate maintenance of granulosa cell, and *Hif1a*, *Creb3I2*, *Zfp71I*, and *Srebf1* play roles in the functions of Theca-G (Supplementary Figs. S5f–i).

Non-proliferating cells in C7–C10 were selected to construct pseudotime trajectory (Fig. 6a). Cells were ordered from the beginning (blue) to the end (red) as they appeared by sampling time in the corresponding UMAP plots (Fig. 6b, c). The result indicated that stroma lineage cells were divided into two routes during ovary development. One portion of stroma lineage cells developed into stromal cells and the others differentiated into Theca-S (Fig. 6b, c). We selected cell cluster-enriched genes and analyzed their dynamic expression along the predicted pseudotime (Fig. 6d). The theca-specific genes (*Cyp17a1, Nr5a1, Hsd3b1*) and the stromal cell-specific gene (*Col4a1*) were gradually increased from E14.5 to P14. In this process, transcription factors *Prrx2, Rarb, Gata2, and Lhx8* were highly activated in cluster 9 and 10, suggesting that these genes might be involved in regulating the differentiation of stromal cells. *Cebpa, Nr5a1, Arntl, Six4, Crem*, and *Zfp931* were abundantly expressed in cluster 10, indicating that these genes might play important roles in Theca-S differentiation or lineage maintenance (Supplementary Fig. S5f–i).

To further explore the relationship among those transcription factors in differentiation or cell fate maintenance of stromal and granulosa lineages, we conducted the regulation network of key transcriptional regulators in Theca-G (Supplementary Fig. S6b) and Theca-S (Supplementary Fig. S6c). Cebpa and Nr5a1 were the key regulators in both Theca-G and Theca-S. In Theca-G, *Cebpa* controlled the expression of *Txndc11*, while the key target of *Nr5a1* was *Nap1I5*. *Fasn* was the only common target of *Nr5a1* and *Cebpa* in Theca-G. In contrast, more common targets of *Nr5a1* and *Cebpa* (*Hsd3b1, Cyp17a1, Wnt6,* et al.) were identified in Theca-S.

## Discussion

Ovarian morphogenesis is a highly orchestrated process in which ovarian follicles are formed through intricate communication between germ cells and somatic cells. Defects in this process lead to reproductive disease and infertility in females^26,27^. *Foxl2* has been considered a granulosa-specific maker gene and the FOXL2-positive somatic cells in fetal ovaries will differentiate into granulosa cells in developing ovarian follicles. In this study, we found that FOXL2-positive cells in fetal gonads give rise to three different cell types, including granulosa cells, theca-interstitial cells, and ovarian stromal cells. Theca-interstitial cells are steroidogenic cells. They express enzymes for the biosynthesis of steroid hormones. However, the location and morphology of these cells are different. Theca cells are typical squamous epithelial cells and are separated from granulosa cells by a basal membrane^27^. In contrast, interstitial cells are stroma-like cells located in the mesenchyme between ovarian follicles.

Although the morphology and location of theca and interstitial cells are different, whether the origin and functions of these cells are different have not been investigated previously. In this study, we demonstrated that theca and interstitial cells are derived from different cell lineages. Both Theca and interstitial cells are derived from FOXL2-positive cells in fetal ovaries. However, Theca are closely related to granulosa cells lineage and directly differentiates from granulosa cells, so we called these cells Theca-G. By contrast, interstitial cells come from stromal cells, which are separated from granulosa cells lineage at an early developmental stage, then we called them Theca-S. Although our study demonstrated that steroidogenic cells in ovaries were derived from *Foxl2*-expressing cells, we could not exclude the possibility that the cells from mesonephros and other sources also contribute to theca-interstitial cell lineage.

CYP17A1 is a cytochrome P450 enzyme with 17-alpha-hydroxylase and C17, 20-lyase activities, which converts pregnenolone and progesterone to dehydroepiandrosterone (DHEA), cortisol, testosterone, and estradiol^28,29^. The results of single-cell transcriptomics showed that the 3β-HSD-positive cells derived from granulosa cell lineage were negative for CYP17A1, whereas the steroidogenic cells derived from stromal cell lineage expressed CYP17A1. These results were further confirmed using *Cyp17a1-CreER*^*T2*^ and *mT/mG* reporting mice. Our results showed that CYP17A1 was expressed in Theca-S, not in Theca-G, which was consistent with the previous study^30^. These results indicate that the gene expression profile is different between Theca-S and Theca-G. Whether the functions between these two cell types is also different is unclear, which needs further investigation.

The reconstruction of the *Foxl2*-expressing cell lineages in the developing XX gonad allowed us to identify transition states leading to the differentiation of the granulosa cells, theca-interstitial cells, and the stromal cells from a common progenitor cell population. Several intermediate stages of granulosa cells were identified by single-cell transcriptomics analysis. *AMH* is a granulosa cell-specific gene and first expresses in the granulosa cells of primary follicles^19^. As expected, its expression was significantly increased in granulosa lineage from E14.5 to P14. Interestingly, we also found *Arxes* and *Klf4* were gradually reduced from E14.5 to P14. The expression of *H2-D1* was increased first and then decreased along with granulosa cell differentiation, suggesting that these genes are also involved in the lineage specification of granulosa cells.

**Fig. 7:**
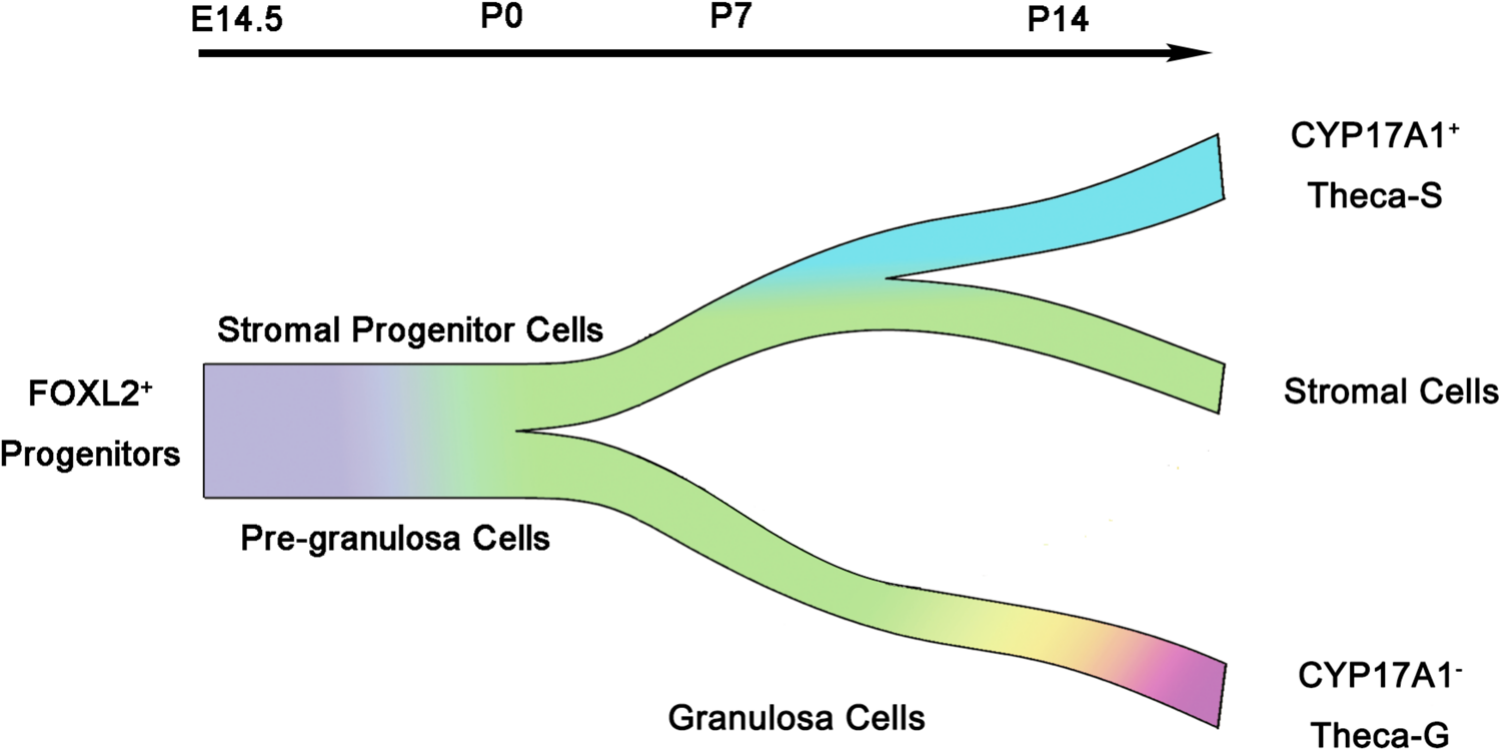
Schematic image of the FOXL2^+^ cell lineage reconstruction during ovary development. FOXL2^+^ progenitor cells in the fetal ovary give rise to stromal cells, CYP17A1^+^ Theca-S, granulosa cells, and CYP17A1^−^ Theca-G. A subset of FOXL2^+^ progenitor cells acquire stromal cell fate at P0, and then a small portion of these early stromal cells gradually differentiate as CYP17A1^+^ Theca-S between P7 and P14. The rest of the progenitor cells differentiate as pre-granulosa cells at P0. Pre-granulosa cells maintain their granulosa cell fate and become granulosa cells, and then CYP17A1^−^ Theca-G are gradually derived from granulosa cells.

In this study, we demonstrated that *Foxl2*-expressing somatic cells in fetal ovaries gave rise to granulosa cells, theca-interstitial cells, and stromal cells. Theca-S and Theca-G are derived from different progenitor cells. CYP17A1-negative Theca-G cells were directly differentiated from granulosa cells, whereas CYP17A1-positive Theca-S were derived from ovarian stromal cells, which were separated from granulosa lineage at P0 (Fig. 7). We also found the gene expression between Theca-S and Theca-G was different. Our study provides an important resource for better understanding somatic cell differentiation in ovary development.

## Methods

### Animals

The mice were maintained in the specific pathogen-free animal facility of the Experimental Animal Center of the Institute of Zoology, Chinese Academy of Sciences, with a humidity of 35% ± 4%, a stable temperature of 24 ± 1 °C, and 12/12 h light/dark cycle. All the animal experiments were performed under the guidelines of the Animal Research Committee of the Institute of Zoology, Chinese Academy of Sciences. *mT/mG; Foxl2-Cre* female mice were obtained by crossing *mT/mG* mice^18^ with Foxl2-Cre mice^17^. *mT/mG* reporting mouse is a double-fluorescent Cre reporter mouse, which expresses membrane-targeted tandem dimer Tomato (mT) prior to Cre-mediated excision and membrane-targeted green fluorescent protein (mG) after activation of cyclization recombination enzyme (Cre).^18^ *mT/mG; Foxl2-CreER*^*T2*^ mice were obtained by crossing *mT/mG* mice with *Foxl2-CreERT2* mice^31^. *mT/mG; Cyp17a1-CreER*^*T2*^ mice were obtained by crossing *mT/mG* mice with *Cyp17a1-CreERT2* mice. The genotype was examined by PCR using DNA isolated from tail tips. The primer sequence used were as follows:

*Foxl2*-Cre-Forward, 5′ -GAGAAGAGAGTGAGAGCCGC-3′,

*Foxl2-*Cre-Reverse, 5′-GCCAGCAGGGTCCCCGCCGTGTCT-3′,

*Foxl2-*CreER^T2^-Forward, 5′-TCCAATTTACTGACCGTACACCAA-3′,

*Foxl2*-CreER^T2^-Reverse, 5′-CCTGATCCTGGCAATTTCGGCTA-3′,

*Cyp17a1-*CreER^T2^-Forward, 5′-TGGCCCGGCAGGAGCTCTTTATCTTCAT-3′

*Cyp17a1*-CreER^T2^-Reverse, 5′-ACAGGGAGGGCAGGCAGGTTTTGGTG-3′

*mT/mG*-Forward, 5′- CTCTGCTGCCTCCTGGCTTCT-3′,

*mT/mG*-Reverse1, 5′-CGAGGCGGATCACAAGCAATA-3′,

*mT/mG*-Reverse2, 5′- TCAATGGGCGGGGGTCGTT-3′.

### Immunofluorescence

The ovaries were fixed in 4% paraformaldehyde, dehydrated in series ethanol, and embedded in paraffin. About 5-μm sections were prepared by microtome, dewaxed in dimethylbenzene, and re-hydrated in series ethanol. Sections were blocked for 1 h at room temperature in 5% BSA with 0.3% TritonX-100. After blocking, sections were incubated with primary antibodies diluted in 1% BSA for 1 h at room temperature. Then, FITC or TRITC conjugated secondary antibodies were employed for 1 h at room temperature and DAPI were used to stain nuclei for 15 min. Primary antibodies used are listed in the following table. Immunolabelled sections were imaged with a confocal laser scanning microscope (Zeiss 880).

**Table:** 

Antibody	Catalog	Dilution	Company
anti-3β-HSD	sc-30820	1:400	Santa Cruz
anti-SOX9	AB5535	1:200	Chemicon/Millipore
anti-WT1	ab89901	1:400	Abcam
anti-GFP	sc-9996	1:100	Santa Cruz
anti-FOXL2	ab5096	1:100	Abcam
anti-RFP	600-401-379 S	1:1000	Rockland

### Isolation of GFP-positive cells and single-cell RNA sequencing

The gonads from *mT/mG; Foxl2-CreER*^*T2*^ female mice were collected at E14.5, P0, P7, and P14, and digested with trypsin and collagenase for 5–8 min at 37 °C to obtain single-cell suspension. The cell suspension was filtered through a 40-μm cell strainer to remove undigested cell clusters. GFP-positive cells were sorted by FACS. Single-cell libraries were constructed using the 10 × single-cell 3′ Library & Gel Bead Kit v2 according to the manufacturer’s instruction^32^. In short, cell counts were assessed with a hemocytometer (Luna-fl, Logos Biosystems) and the cell concentration was adjusted to about 600 cells/μL. All cells from E14.5 gonads were added to the channel of the 10× chip. For other stages, 12,000 cells were added to each channel, and about 7000 cells were captured. Captured cells were lysed, and the released RNA was barcoded through reverse transcription. Then, the cDNA was amplified for the library construction, and the qualities of cDNA and cDNA libraries were assessed by Agilent 2100. Finally, libraries were sequenced on an Illumina Hiseq X Ten platform (CapitalBio, Beijing).

### 10× Genomics data pre-processing

Reads processing was performed using the 10× Genomics Cell Ranger (3.1.0) workflow^32^ with default mapping arguments. Briefly, the cellranger count command line was used to assign barcodes, map reads, and quantify UMI for each sample. Reads were mapped to the mm10 genome (including eGFP sequence) and counted with GRCm38.84 annotation (including eGFP information). The cellranger aggr command line was executed to uniform the sequencing depth for all 10× samples in this study, and the mean reads per cell were more than 30,000 post-normalization.

### Filtering of the scRNA-seq Data

For all sequenced single cells, we first filtered out the cells with a percentage of mitochondria genes of more than 10%. Then Scrublet python package was exploited to compute multiple cells for each sample^33^. All samples were used to create the Seurat object^34^, and only genes expressed in more than three cells were retained. After further excluding the doublet cells, low-level GFP expressing cells (including germ cells and macrophages), and cells with low quality (cells with fewer than 500 genes or the percentage of mitochondria genes less than 1%), 23,534 cells remained to do the following analysis.

### Clustering, DEG, and GO analysis

Single-cell data were analyzed with the Seurat v3 R package^34^. 23,534 cells were used to create the Seurat object, and only genes expressed in more than three cells were retained. Variable genes were computed with the “mean.var.plot” method. “Elbow Plot” was used to determine an appropriate number of dimensions to perform the nonlinear dimensional reduction (UMAP). Unsupervised clustering analysis was performed by the “Find Clusters” function to identify cell clusters. The Seurat “Find all Markers” function was used to compute DEGs between cell clusters. The biological process aspect of GO analysis was performed using the clusterProfiler R package^35^.

### PAGA analysis

The Seurat object was converted to a loom file by the as.loom function of loomR package^34^, then the loom file was loaded to the python environment by the sc.read_loom function of Scanpy python package^36^. After setting the neighbor argument, the relationship between different cell clusters was evaluated by the partition-based graph abstraction (PAGA) analysis with the Scanpy python package^36^.

### Pseudotime analysis

The Monocle2 R package^37^ was used to compute the developmental pseudotime of granulosa and stromal lineage cells, respectively. After selecting the cells of the individual lineage, the UMI count data and metadata were exported from the Seurat3 object. The New Seurat2 object was built based on the UMI count data and metadata, and only the genes expressed in more than three cells were retained. The Seurat2 object was then converted to the Monocle2 object by the importCDS function of Monocle2^37^. Variable genes computed by the “mean.var.plot” method of seurat package were used to do the unsupervised ordering of the cells with default settings of Monocle2 R package^37^.

### SCENIC analysis

SCENIC analysis was carried out following the SCENIC command line protocol^38^. SCENIC command line version was used to do gene regulatory network inference, regulon prediction, and cellular enrichment (Area Under the Curve, AUC) processes with the filtered 23,534 GFP^+^ cells. SCENIC UMAP was computed based on the AUC matrix. Regulon specificity scores (RSS) were computed based on the cell clusters identified by Seurat. We chose the top regulons for each cell cluster. Finally, the SCENIC AUC heatmap was plotted with reported regulons and top regulons of each cell cluster by the pheatmap R package.

## Supplementary information

Supplemental information

## Data Availability

All data generated or analyzed during this study are included in this article and its supplementary information files. The data reported in this paper were available at the NCBI Gene Expression Omnibus (GEO) with the accession GSE163879. The softwares used in this paper were Seurat, Monocle2, clusterProfiler, pheatmap R packages, and Scrublet, Scanpy, and SCENIC python packages.
