# Supplementary material for: Dissecting the fate of Foxl2-expressing cells in fetal ovary using lineage tracing and single-cell transcriptomics
Source: Cell Discov. 2022 Dec 27;8:139. doi: 10.1038/s41421-022-00492-1 (PMC9794781; doi:10.1038/s41421-022-00492-1)
Supplement: Supplementary file 1 — Supplemental information [file 41421_2022_492_MOESM1_ESM.docx]

**Supplemental information**


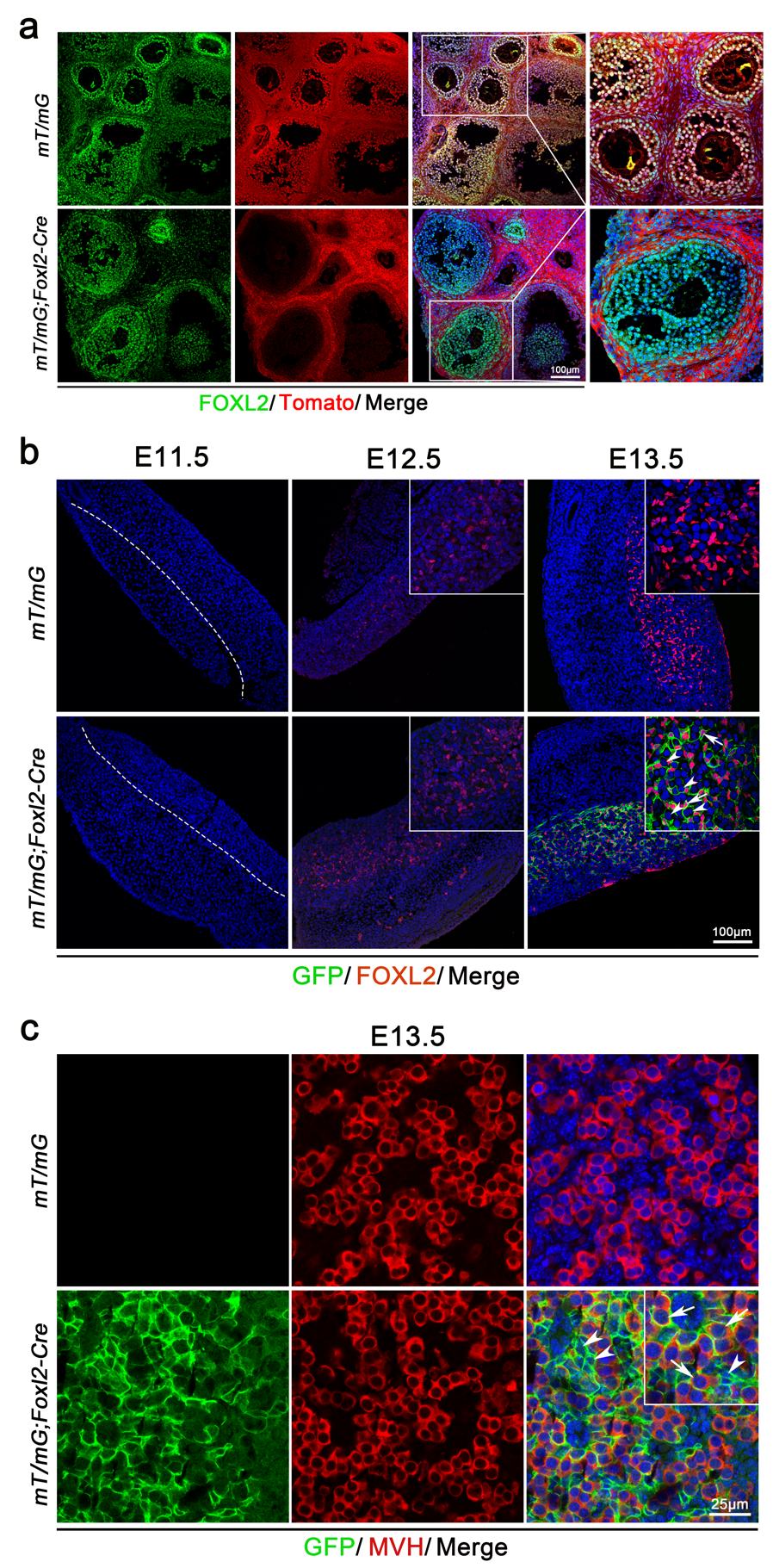


Supplementary Fig. S1 **a**. Co-immunofluorescence of Tomato and FOXL2 in ovaries of *mT/mG; Foxl2-Cre* mice at P14. In *mT/mG* ovaries, all the cells were Tomato positive (red), including FOXL2-positive (green) granulosa cells (top panel). In *mT/mG; Foxl2-Cre* ovaries, no Tomato signal (red) was detected in FOXL2-positive (green) granulosa cells (bottom panel). **b**. Immunostaining of FOXL2 and GFP *in mT/mG; Foxl2-Cre* mice at E11.5, E12.5, E13.5. GFP signal was not detected in the ovaries of *mT/mG* mice at E11.5, E12.5, E13.5 (top panel). GFP signal was not detected in the ovaries of *mT/mG; Foxl2-Cre* mice at E11.5, E12.5*.* GFP signal was detected at E13.5(inset, white arrows and arrowheads). **c**. Germ cells were labeled with MVH (red), and no GFP signal was detected in *mT/mG* ovaries (top panel). In *mT/mG; Foxl2-Cre* mice. some MVH positive germ cells were surrounded by GFP signal (inset, white arrows).


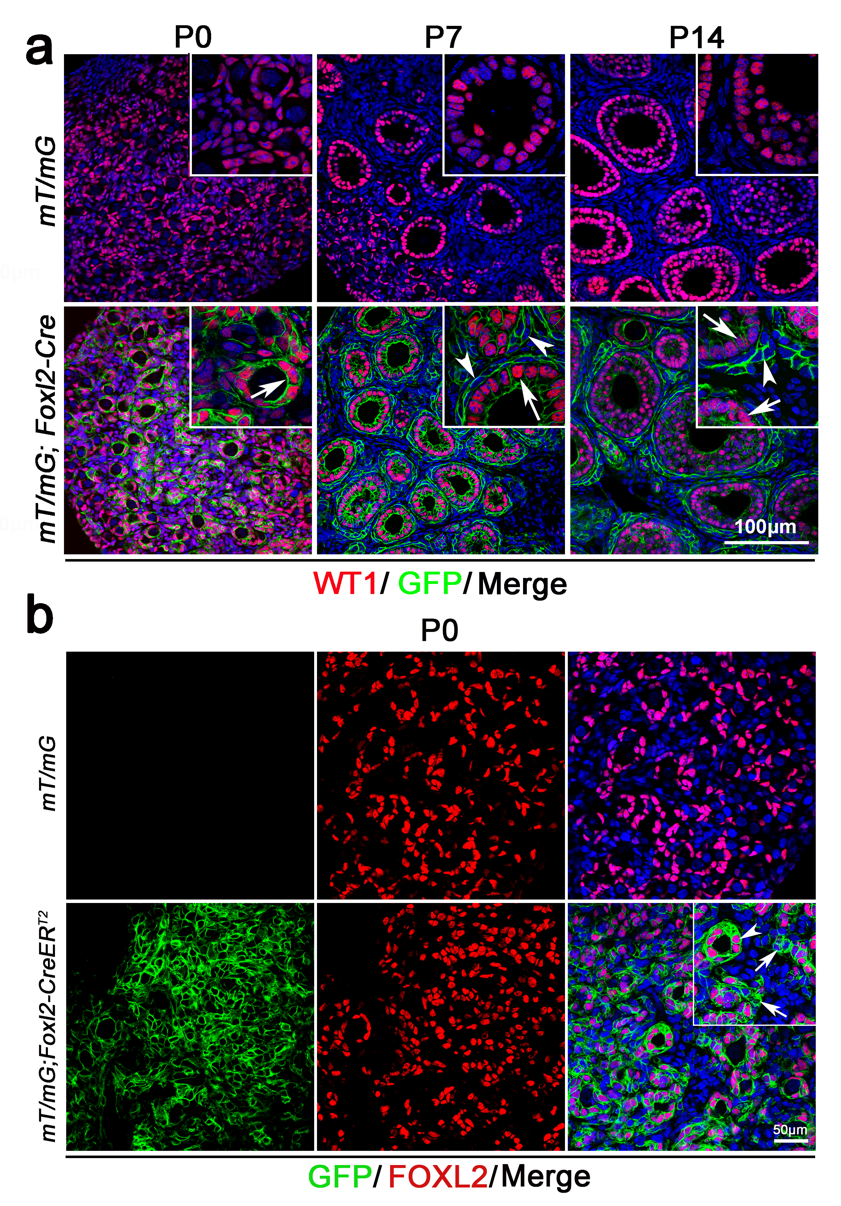


Supplementary Fig. S2 **a**. Lineage tracing of *Foxl2*-expressing cells in ovary with *Foxl2-Cre* mice. *Foxl2-Cre* mice were crossed with *mT/mG* reporting mice and GFP signal was examined by immunostaining. GFP signal was not detected in the ovaries of *mT/mG* mice at P0, P7, and P14 (top panel). GFP signal was detected in the ovaries of *mT/mG; Foxl2-Cre* mice at P0, P7, and P14 (bottom panel) and GFP signal was detected not only in WT1-positive granulosa cells (inset, white arrows), but also in theca-interstitial cells (inset, white arrowheads).

**b**. Lineage tracing of *Foxl2*-expressing cells in fetal ovary with *Foxl2-CreER^T2^* mice at P0. *mT/mG; Foxl2-CreER^T2^* mice were induced with tamoxifen at E13.5 and GFP signal was examined by immunostaining at P0. No GFP signal was detected in the ovaries of *mT/mG* mice when Cre was induced with tamoxifen at E13.5(top panel). GFP signal was detected in FOXL2-positive cells (bottom panel, inset, white arrowheads) and FOXL2-negative cells (bottom panel, inset, white arrows).


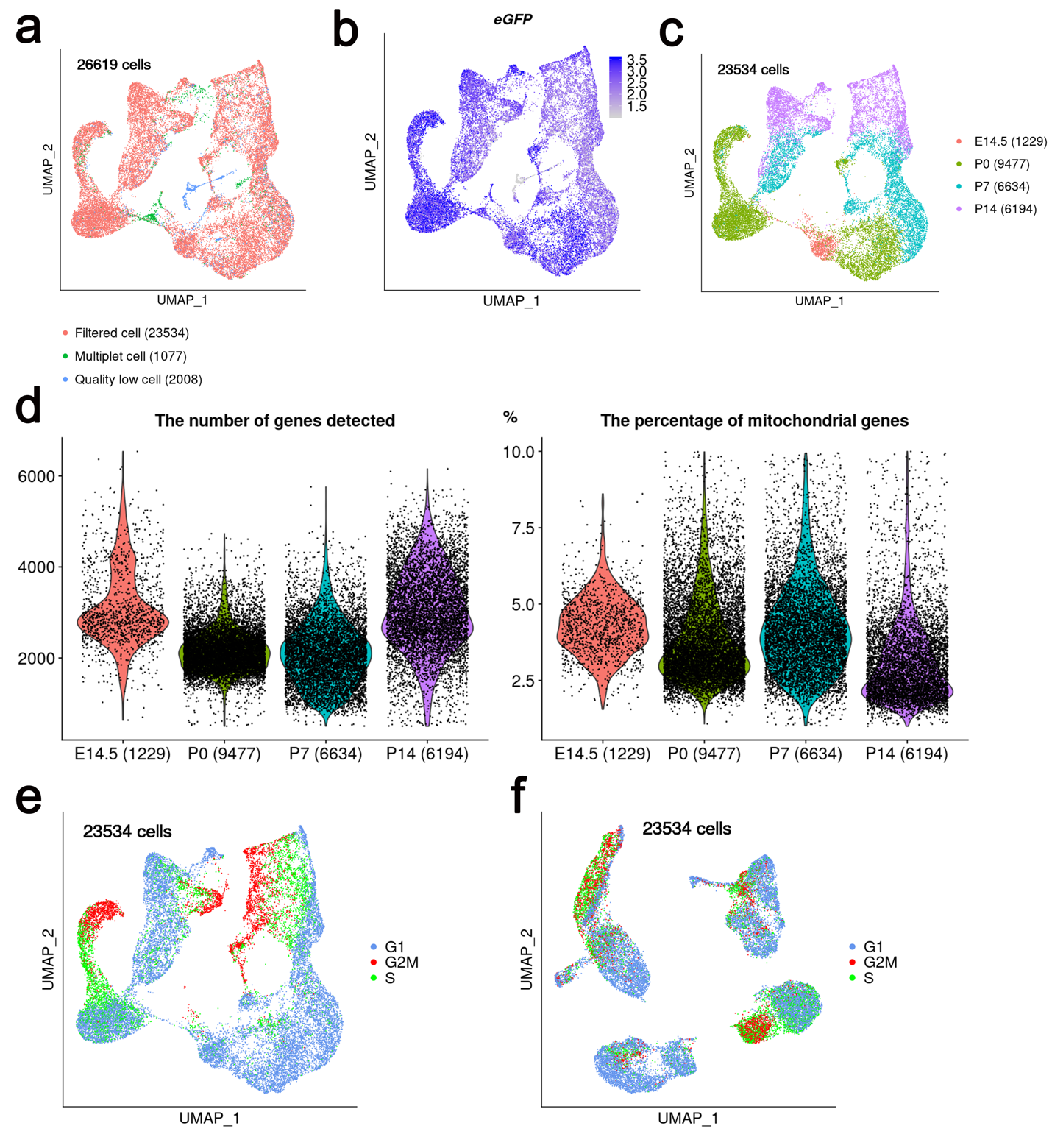


Supplementary Fig. S3 The filtering of GFP^+^ cells with high quality.

**a.** Expression matrix-based Uniform manifold approximation and projection (UMAP) plot showed all the captured cells (26619 cells) with more than 500 genes expressed, and the percentage of mitochondrial genes in each cell was between 1% and 10%. Cells were colored by their characters as indicated.

**b.** UMAP plot of GFP expressing cells. The colors from grey to blue indicated low to high level of gene expression.

**c.** UMAP plot of cells after filtering the multiplet and low quality cells, with cells colored by time points of sample collection.

**d.** Violin plots showed the number of genes detected (left) and the percentage of mitochondrial genes (right) per cell.

**e.** UMAP plot with cells colored by cell cycle phases.

**f.** UMAP plot was rebuilt from panel **E** after mitigating the effects of cell cycle heterogeneity by Seurat R package**,** with cells colored by cell cycle phase.


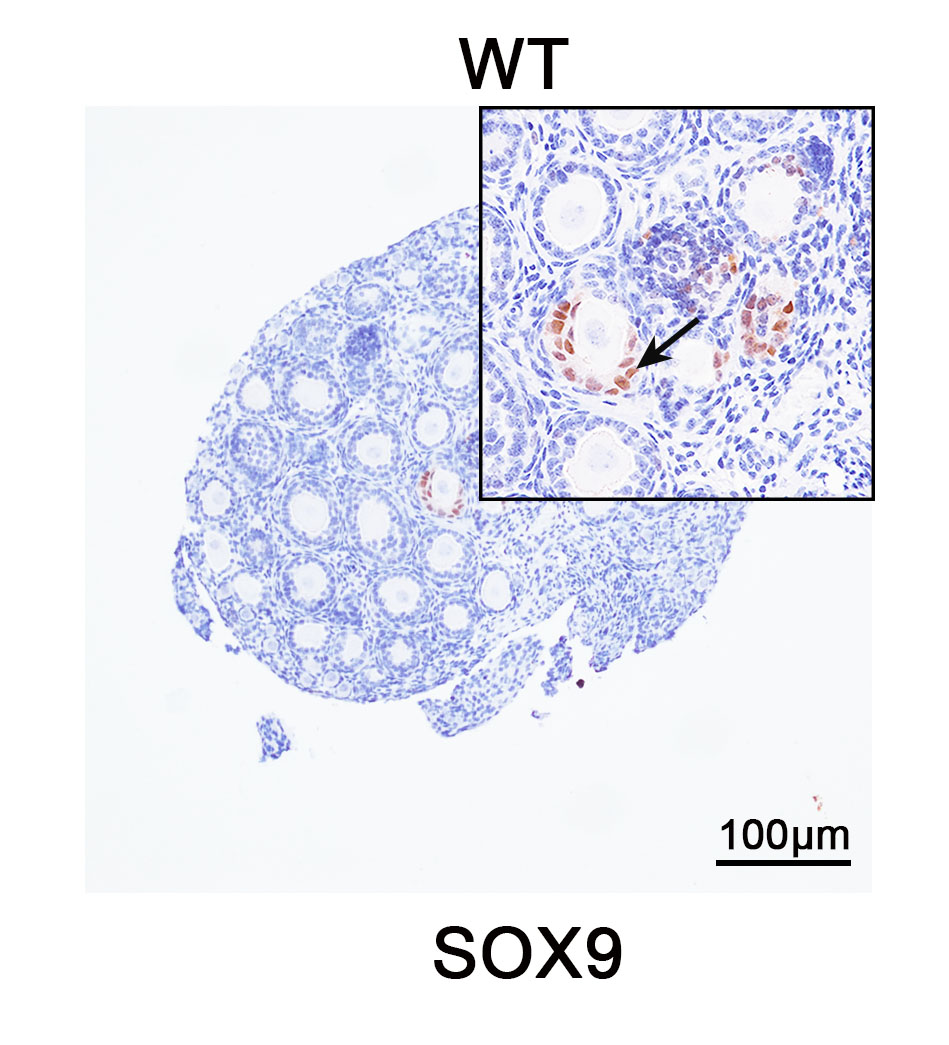


Supplementary Fig. S4 SOX9 positive cells were detected in P7 ovaries. SOX9 were detected in P7 ovaries of wild-type (inset, black arrow).


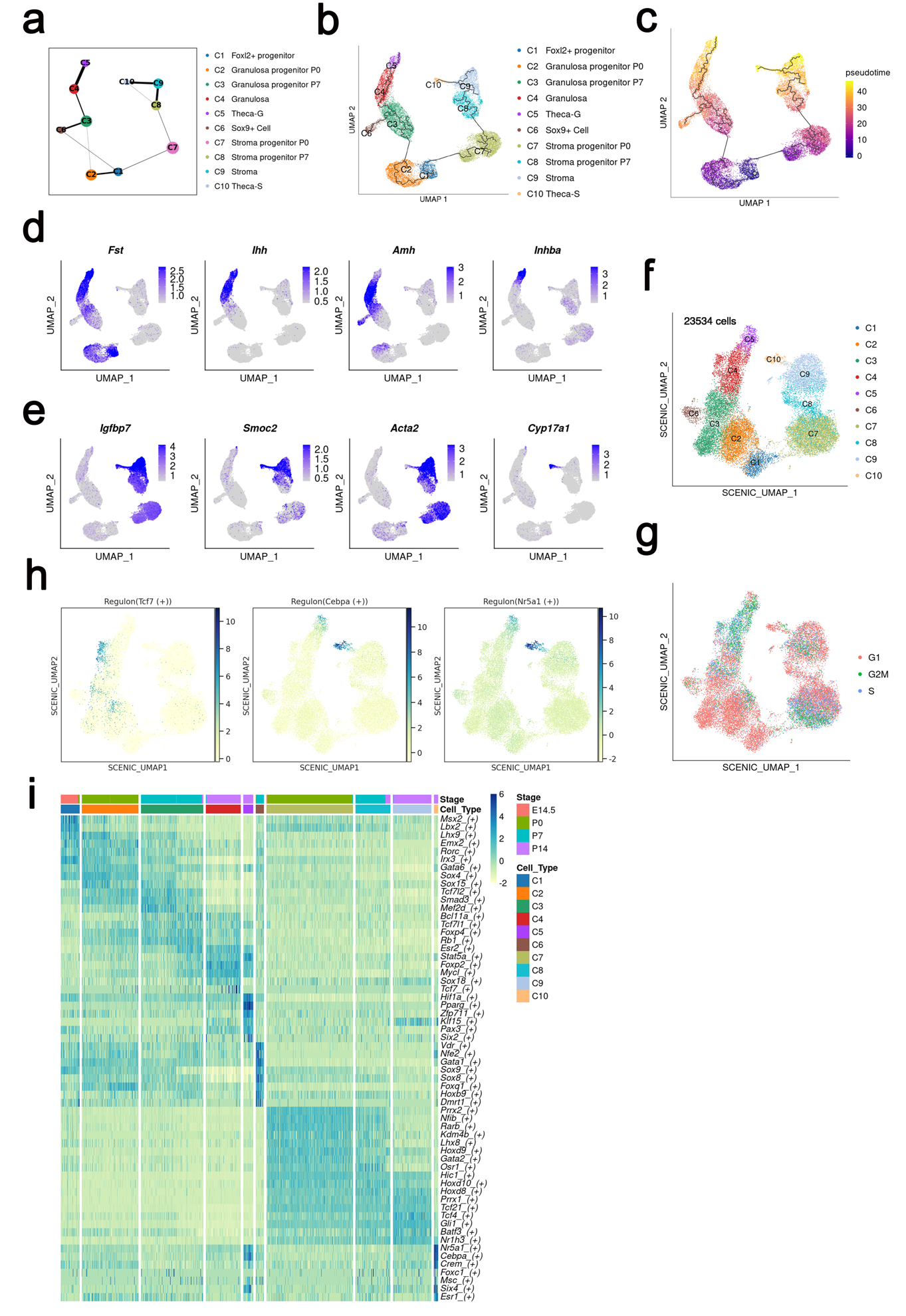


Supplementary Fig. S5 Visualization of the progeny of *Foxl2*-expressing cells and SCENIC analysis of *Foxl2*-expressing cells during ovarian development.

**a.** Partition-based graph abstraction (PAGA) summarizing the relationships between the cell clusters as shown in panel **b**. Nodes corresponded to the cell clusters, and edges reflected the confidence of adjacency between clusters (thicker edges indicate higher confidence). Node sizes increased as a function of the number of cells within each cluster.

**b.** Single-cell trajectories were constructed on UMAP plot by monocle3 R package, and cells were colored by cell clusters.

**c.** Pseudotime ordering of cells shown in panel **a**, with cells colored by pseudotime. Color of pseudotime from dark violet to yellow represented the cell state from naïve to mature.

**d**. UMAP plots as shown in panel **b** showing the highly expressed genes in granulosa and Theca-G cells.

**e**. UMAP plots as shown in panel **b** showing the highly expressed genes in stroma and Theca-S cells.

**f.** SCENIC Regulon matrix-based UMAP plot showed the cells (25234 cells) and cell clusters in **Fig 3b.**

**g.** UMAP plot as shown in panel **a**, with cells colored by cell cycle phases.

**h.** UMAP plot as shown in panel **f** showed the activities of TF *Tcf7*, *Cebpa*, and *Nr5a1.*

**i.** Heat map showed the activities of representative transcription factors (TFs) in Foxl2^+^ progenitor derived cells (25234 cells). The color key from yellow to blue indicated low to high TF activity. Cell clusters and sampling time points were indicated at the top of heatmap.


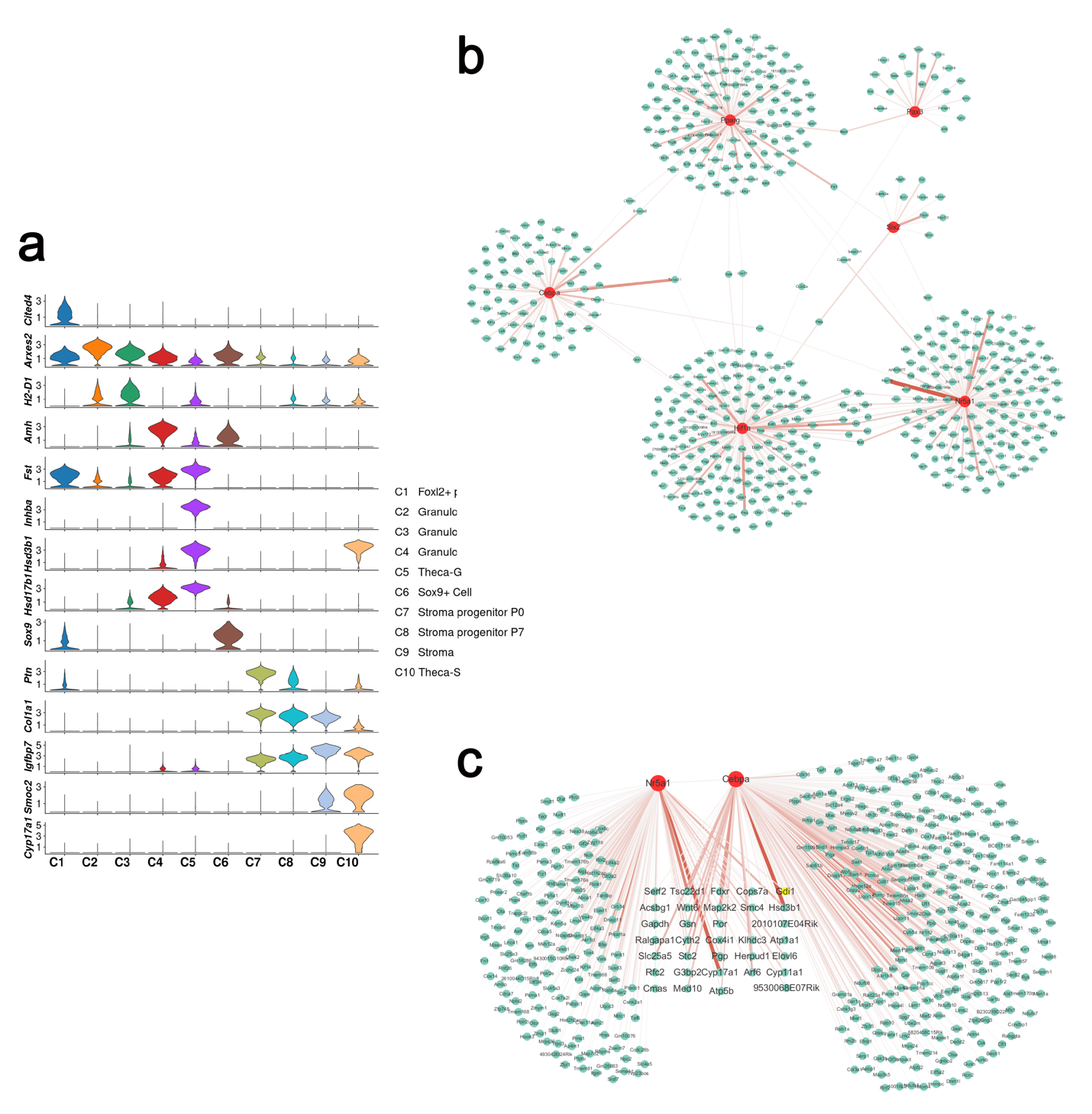


Supplementary Fig. S6 Highly expressed genes in 10 cell clusters and transcription network for the differentiation of Theca-G and Theca-S cells.

**a.** Violin plots of highly expressed genes in 10 cell clusters.

**b.** Regulatory network visualized potential key transcriptional regulators in Theca-G cells (C5). Top 6 TFs were colored in red and target genes were in green. Only the TF-target connected with a high importance (>2) was retained to construct the network, and the edge width indicated the TF-target importance.

**c.** Regulatory network visualized potential key transcriptional regulators in Theca-S cells (C10). Top 2 TFs were colored in red and target genes were in green.
